# Supplementary figures and images for: Comparing metabolic engineering scenarios using simulated design-build-test-learn-cycles
Source: Front Bioeng Biotechnol. 2026 Jun 26;14:1802948. doi: 10.3389/fbioe.2026.1802948 (PMC13351528; doi:10.3389/fbioe.2026.1802948)

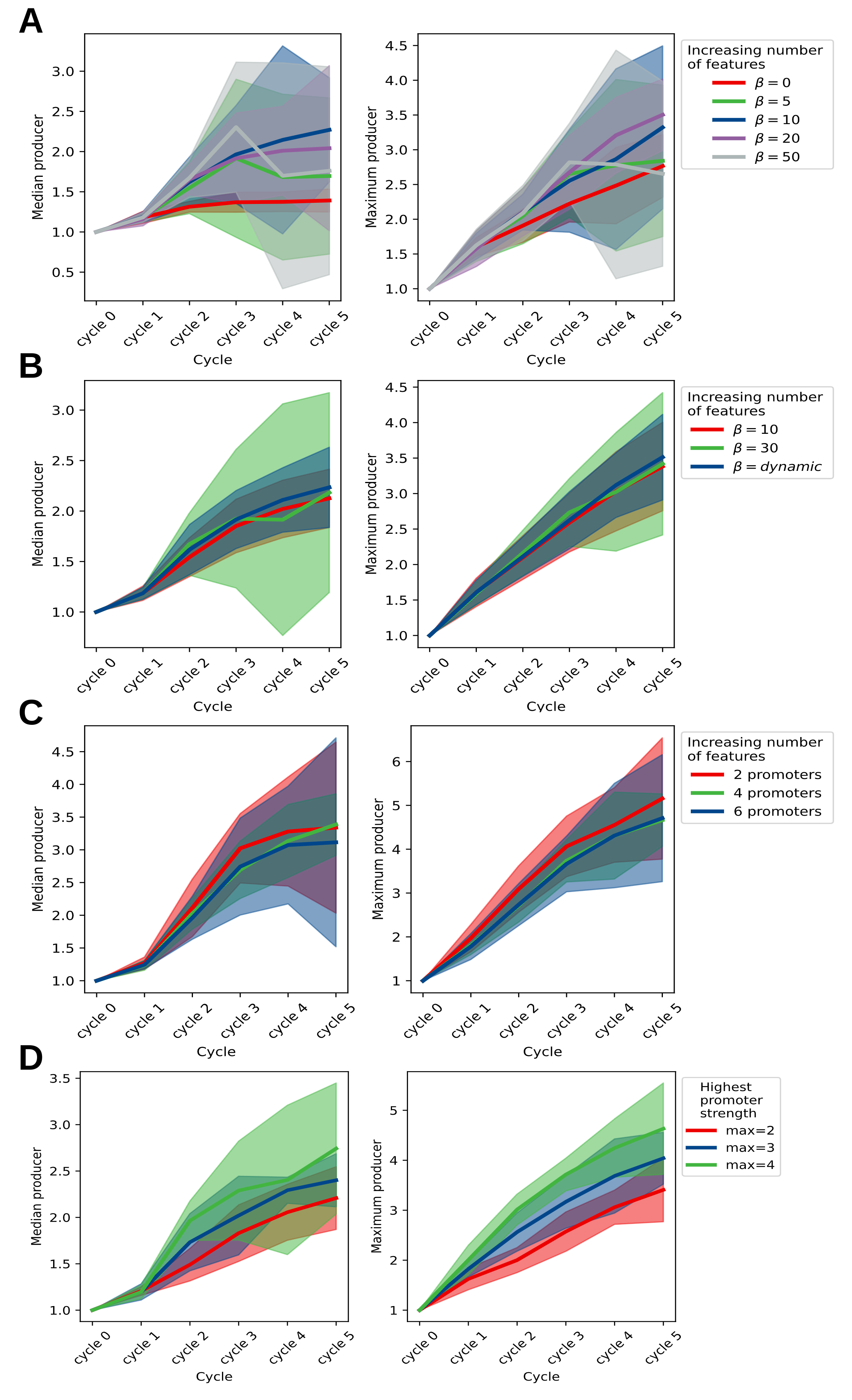

Supplement: Supplementary file 2 [file Image1.tiff]

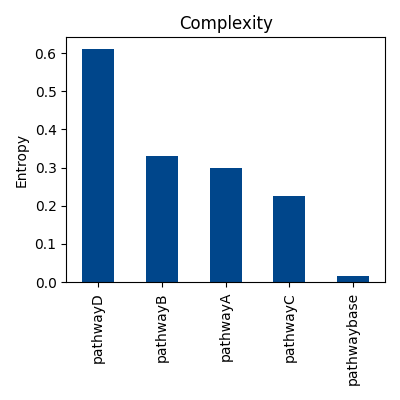

Supplement: Supplementary file 4 [file Image2.tiff]

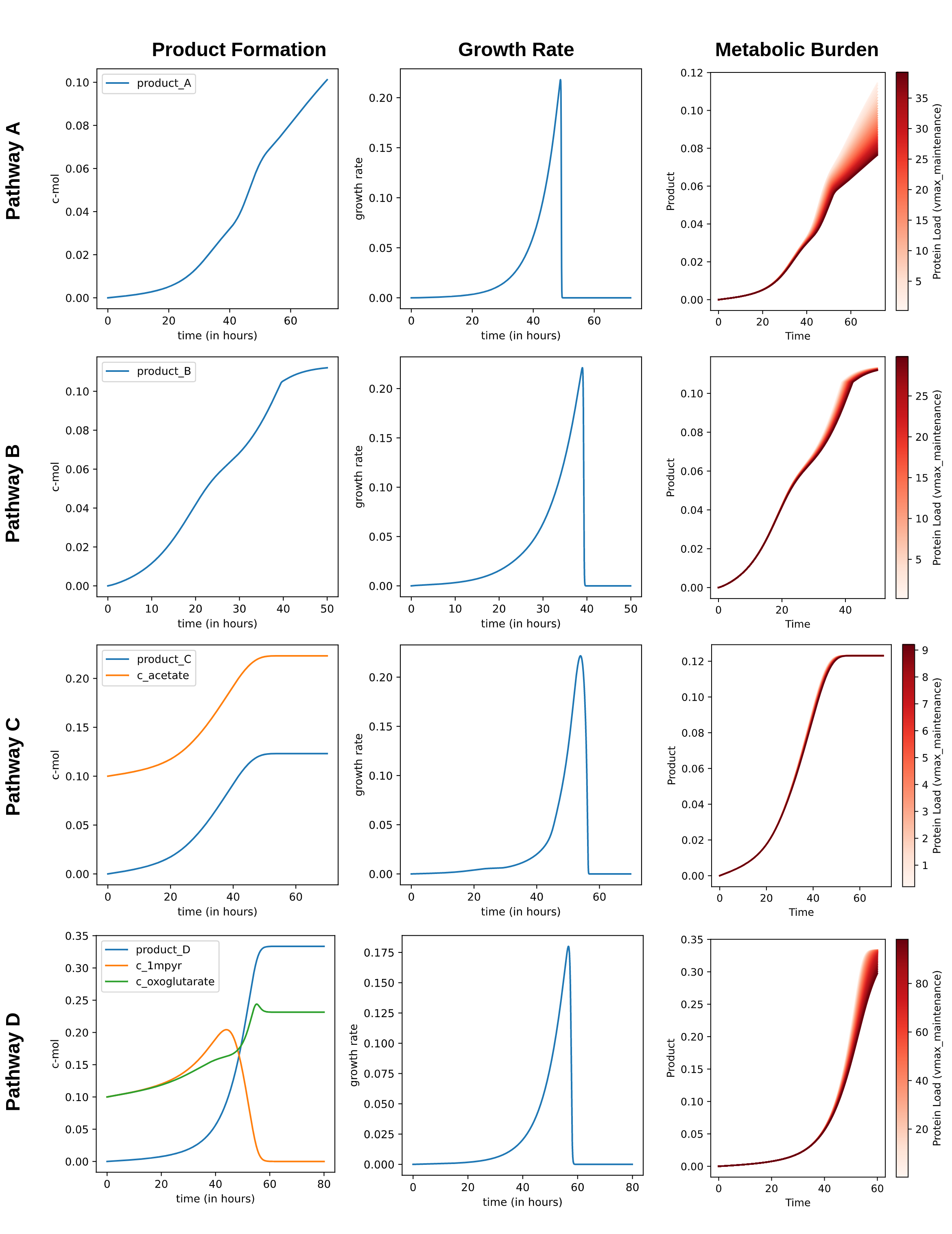

Supplement: Supplementary file 5 [file Image4.tiff]
