# Supplementary material for: Comparing metabolic engineering scenarios using simulated design-build-test-learn-cycles
Source: Front Bioeng Biotechnol. 2026 Jun 26;14:1802948. doi: 10.3389/fbioe.2026.1802948 (PMC13351528; doi:10.3389/fbioe.2026.1802948)
Supplement: Supplementary file 3 [file DataSheet1.pdf]

Supplementary Material for article Comparing metabolic engineering scenarios using simulated design-build-test-learn-cycles

0.1 Pathway Overview

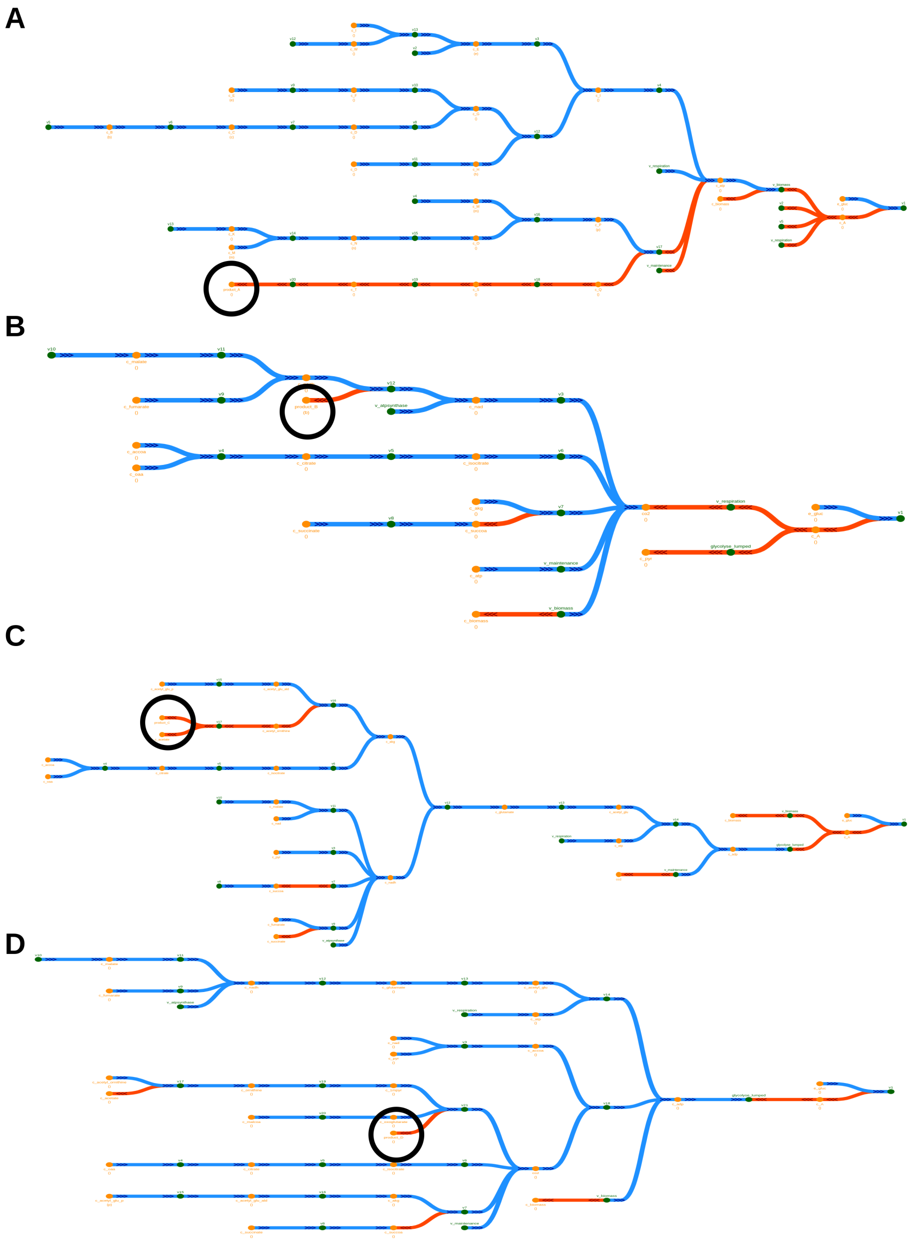

Figure S1. Simplified schematic overview of the four pathway models. The black circles show the optimization targets that we aim to optimize. Fluxer represents metabolic pathways as trees for visualization purposes and therefore does not show topological cycles Hari and Lobo (2020).

0.2 Model validation

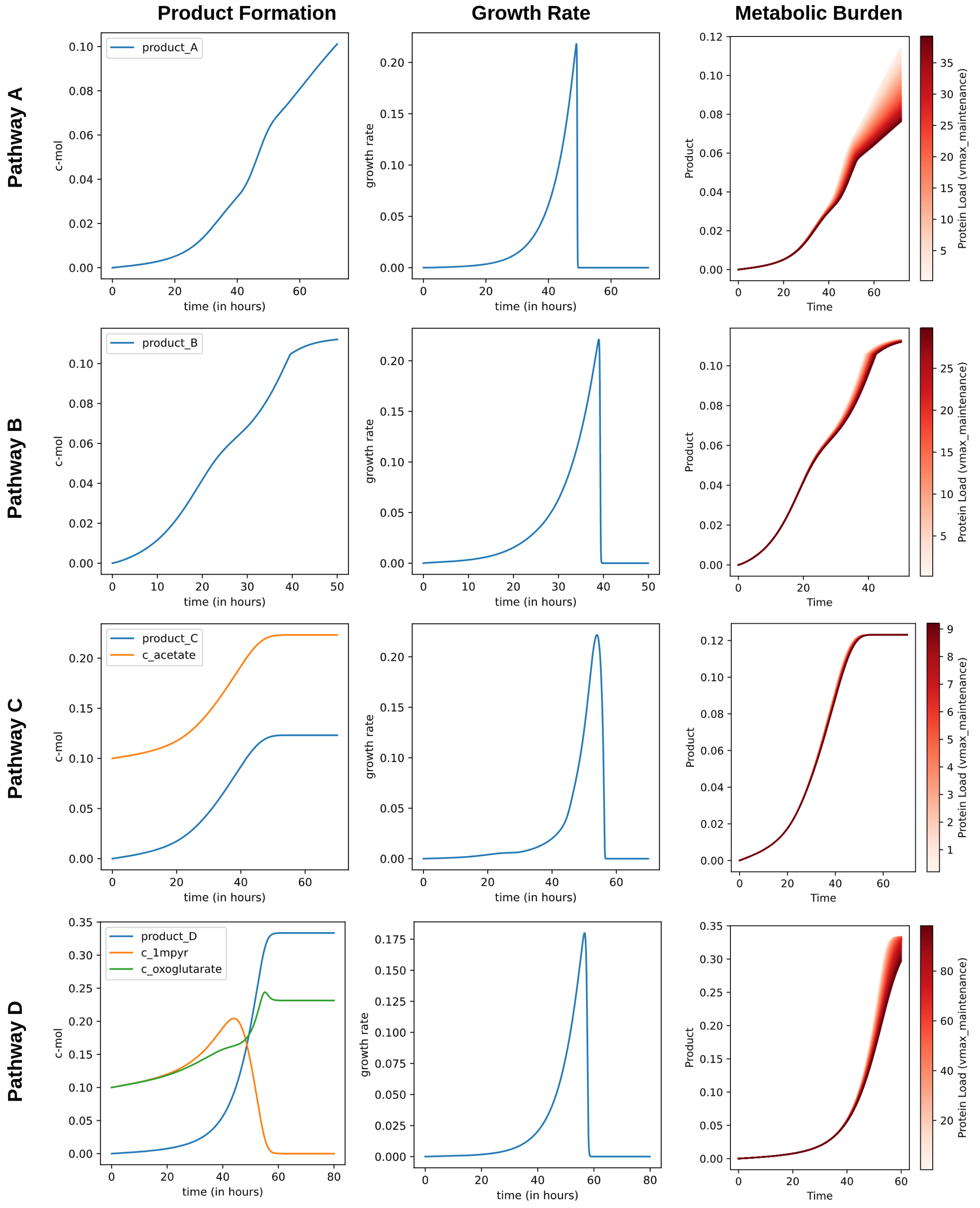

Figure S2. Plots of some characteristics of the bioprocess models. Left) Product formation in the four bioprocess models. Pathway C and D have byproducts. **Middle)** The growth rate for the four pathway models. **Right)** Effect of protein load on the four pathway models

0.3 Additional plots for pathway model A DBTL cycle parameters

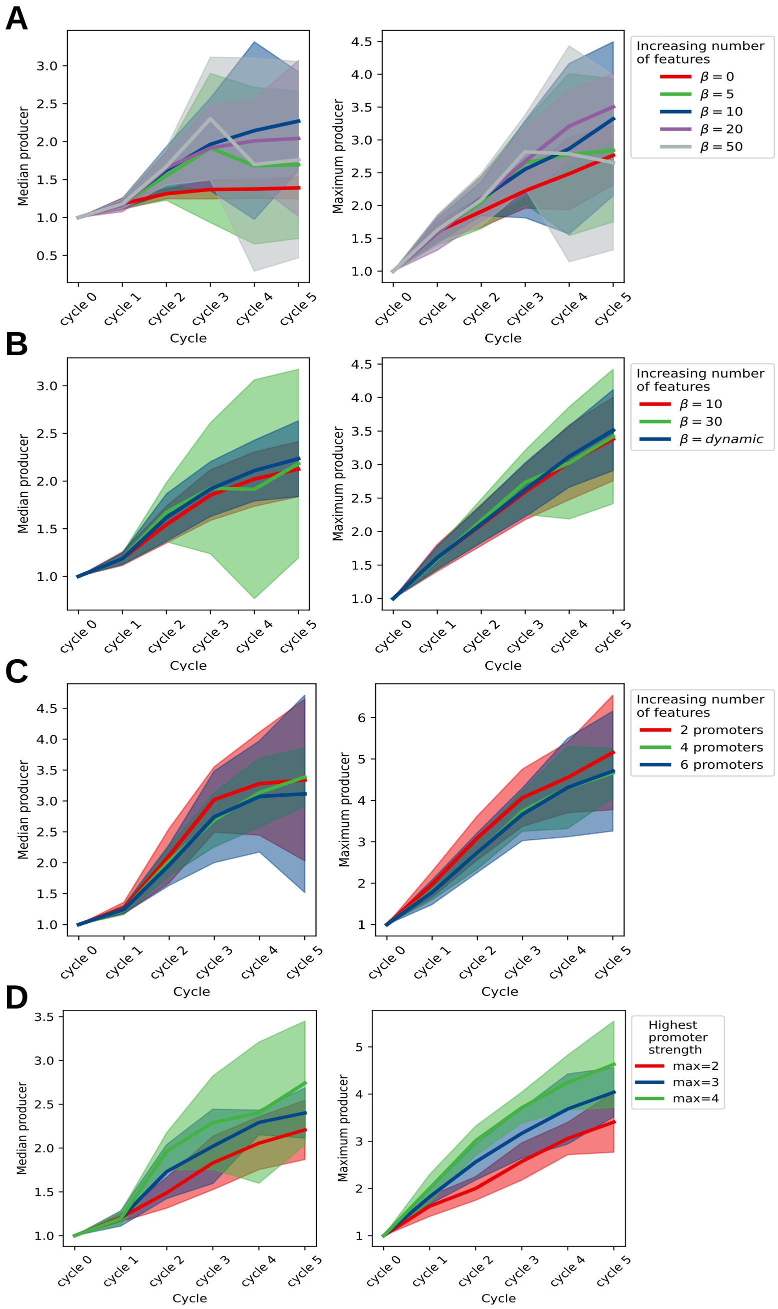

Figure S3. Additional simulated scenarios for pathway model A. **A)** The exploration-exploitation parameter β and the effect on the median and maximum strain performance. **B)** Dynamically balancing this parameter does not improve optimization performance significantly. **C)** Number of promoters used in the library design, on the range 0-2. **D)** Scenario where the strongest expression strength is two, three, or four times stronger than the wild-type promoter.

0.4 Pathway Complexity measure

To quantify the topological complexity of the models used in this study, we used a previously established approach Ghavasieh and De Domenico (2024). Briefly, a graph Laplacian is constructed from the stoichiometric matrix S (eq. S1), which defines a random walk of duration τ over the reaction network. The n-th step of this random walk yields a matrix M, from which we compute the density matrix $\rho_\mathcal{T}$ by normalization with respect to the trace of M (eq. S3). The von Neumann entropy of $\rho_\mathcal{T}$ (eq. S4) is then used as a scalar measure of network complexity. The walk length was set to $\tau = 10$, which we verified to be sufficiently long for the random walk entropy to reach a steady state. This procedure provides a consistent metric for comparing the complexity of distinct metabolic networks (Fig. S4). To enable comparisons across a diverse set of metabolic pathways, we constructed multiple topological variants for each pathway.

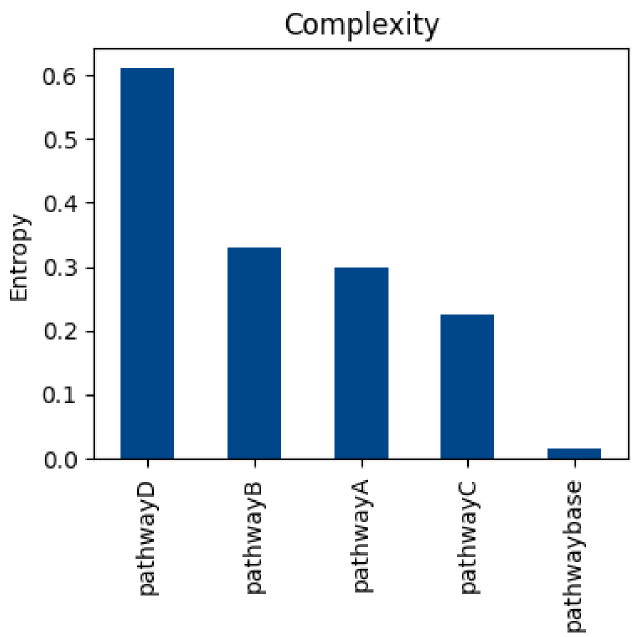

Figure S4. Complexity of the metabolic pathway calculated using the approach taken in Ghavasieh and De Domenico (2024). Pathway model D had the highest complexity associated, while pathway model C was considered the easiest in terms of topology.

$$L = S\,S^T \tag{S1}$$

$$M = e^{\tau L} = \sum_{n=0}^{\infty} \frac{-\tau^n}{n!} L^n \tag{S2}$$

$$\rho_\mathcal{T} = \frac{M}{Tr(M)} \tag{S3}$$

$$H_\mathcal{T} = -Tr(\rho_\mathcal{T} \log(\rho_\mathcal{T})) \tag{S4}$$

REFERENCES

Hari A, Lobo D. Fluxer: a web application to compute, analyze and visualize genome-scale metabolic flux networks. *Nucleic acids research* **48** (2020) W427–W435.

Ghavasieh A, De Domenico M. Diversity of information pathways drives sparsity in real-world networks. *Nature Physics* **20** (2024) 512–519.
